# Exploring the feasibility, acceptability, usability and safety of a digitally supported self-management intervention for uncontrolled asthma: A pre-post pilot study in secondary care

**DOI:** 10.1177/20552076241292391

**Published:** 2024-11-05

**Authors:** Anne Zijp, Anke Versluis, Laura Joosse, Taco Kind, Tess MJ Rouveroy van Nieuwaal, Chantal Wassink-Bergman, Ilonka HPAA van Veen, Els JM Weersink, Niels H Chavannes, Jiska J Aardoom

**Affiliations:** 1 Department of Public Health and Primary Care, 4501 Leiden University Medical Center, Leiden, Netherlands; 2 National eHealth Living Lab, Leiden, Netherlands; 3 SentoMed BV, The Hague, Netherlands; 4 Department of Pulmonary Medicine, Amsterdam University Medical Center, location University of Amsterdam, Amsterdam, Netherlands; 5 Department of Pulmonary Medicine, 3231 Medical Spectrum Twente, Enschede, Netherlands

**Keywords:** Asthma, eHealth, asthma control, asthma self-management, digital supported intervention

## Abstract

**Introduction:**

Asthma control is often suboptimal, which is associated with incorrect medication use and poor asthma management by patients. Astmakompas, a digital self-management intervention, comprises a mobile app for patients, a web portal for healthcare professionals (HCP), and a wireless spirometer. Together, these components are intended to facilitate symptom monitoring, patient-HCP communication and assist in asthma-related self-management.

**Objective:**

The pilot study primarily aims to assess the feasibility, acceptability, usability and safety of Astmakompas from patient and HCP perspectives, and secondarily to explore potential effects on asthma control, medication use, Quality of Life, self-efficacy and (in)direct costs.

**Methods:**

A mixed-method, multi-center, pre-post study design was conducted in two Dutch hospitals, involving patients with uncontrolled asthma and four HCPs. Primary outcomes were assessed post-intervention using questionnaires and in-depth interviews. Secondary outcomes were assessed at baseline and post-intervention using questionnaires, and post-intervention using in-depth interviews.

**Results:**

In general, the data show that Astmakompas was considered feasible, acceptable, usable and safe by both patients (*n *= 14) and HCPs (*n *= 4). Furthermore, qualitative data of 11 patients and all HCPs showed the importance of guidance from HCPs and tailoring of Astmakompas to care needs. Astmakompas helped patients recognise symptoms and provided valuable insights into asthma control while being easy to use. Asthma control improved from baseline (*M *= 2.1, *SD *= 1.3) to post-intervention (*M *= 1.3, *SD *= 1.0, *t*(13) = 2.61; *p *= .02).

**Conclusion:**

Astmakompas shows promise for further implementation and research. Future randomized studies with longer evaluation periods are crucial to assess the tool's impact on asthma outcomes and workload. It is important that HCPs guide and tailor interventions to meet the patient's individual needs and circumstances.

## Background

Asthma is a major chronic disease of the respiratory tract that affects an estimated 262 million people worldwide.^1–3^ Asthma is a substantial source of global economic burden and is ranked 16th among the leading causes of years lived with disability, as well as 28th among the leading causes of burden of disease as measured by disability-adjusted life years (DALYs).^4^ Asthma control is often suboptimal, with approximately 50% of patients having (partially) uncontrolled asthma.^5,6^ These patients have a higher risk of asthma exacerbations, asthma-related hospitalizations, emergency department visits, morbidity and mortality.^7–11^ Poor asthma control is also a predictor of impaired health-related quality of life (QoL).^9,12,13^

Uncontrolled asthma is often associated with the incorrect use of medication and a discrepancy between patients’ perception of their asthma control and actual symptoms. Specifically, patients often perceive their asthma to be better controlled than it is, which can negatively impact medication adherence and in turn affect asthma control.^9,14–16^ The first treatment step for asthma patients is a low dose of inhaled corticosteroids (ICS) for an anti-inflammatory effect on the respiratory tract in combination with long-acting beta-2 agonists (LABA).^17,18^ Still, asthma patients are primarily responsible for their day-to-day asthma management, including symptom monitoring, medication management, adherence to therapy, identifying and managing triggers, and following action plans provided by healthcare professionals (HCPs). Monitoring asthma can be challenging for patients due to factors like the subjective nature of their appreciation of symptoms, variability in symptom presentation, and the potential for underestimating or overestimating symptom severity.

Given that day-to-day asthma management largely rests on the patient, fostering patient empowerment and providing self-management support are essential. Evidence indicates that self-management can improve overall asthma control and can help reduce the frequency of rescue medication.^19,20^ Supported self-management interventions can also help to improve QoL, reduce the use of healthcare resources and contribute to a decrease in indirect healthcare costs.^21–25^

The number of digital applications in healthcare has increased exponentially in recent years.^26^ Several systematic reviews have shown that digital self-management interventions for patients with asthma can have positive effects on asthma control, lung function, and medication use, as well as self-care, self-efficacy, and QoL.^27–29^ Results of a Swedish randomized controlled trial showed that an 8-week app-based self-management program supported by an interface for HCPs improved asthma control compared to conventional treatment in the context of primary care and paediatric care settings.^30^ In addition, a systematic review showed that offering behavioural support through eHealth, when provided more often than once a month, led to improvement in asthma control.^31^ Digital self-management interventions offer advantages for HCPs as well. Such interventions allow HCPs to efficiently deliver health information to patients and gain insights into the course of the disease in patients’ daily lives,^22^ which can ultimately result in more effective and efficient care.^22^

Although the literature shows promising results of (digital) supported self-management interventions in improving asthma control and other important health outcomes, to our knowledge, no research has been conducted on digitally supported self-management interventions in secondary care, nor in the Dutch context specifically. ‘*Astmakompas*’ (‘asthma compass’) is a digitally supported self-management intervention that can be used in secondary care; however, no research has been conducted in this regard. Hence, the primary aim of this pilot study is to examine whether Astmakompas is perceived as feasible, acceptable, usable and safe for adults with asthma from both patient and HCP perspectives. The secondary aim is to explore potential effects on asthma control, medication use, QoL, self-efficacy and direct and indirect costs.

## Methods

### Design and participants

A mixed-method, multi-center, pre-post intervention pilot study was used, with a study duration of 12 weeks. Both quantitative (i.e., questionnaires) and qualitative data (i.e., interviews) were obtained. The study was approved by the Medical Ethics Committee Leiden The Hague Delft (P22.021).

Two populations participated: (1) patients with asthma treated in secondary care and (2) their HCPs including pulmonologists and pulmonary nurses. Both populations were recruited through two hospitals in the Netherlands: Amsterdam University Medical Center, location AMC and Medical Spectrum Twente (MST). In Amsterdam UMC Astmakompas was already part of usual care, whereas, in MST, Astmakompas was used for the first time during this pilot study. Patients were recruited from November 2022 to May 2023.

Eligibility criteria for patients included: (1) being aged 18 years or older, (2) not having used Astmakompas before, (3) having a physician diagnosis of asthma, (4) having uncontrolled asthma as defined by a score of ≥1.5 on the Asthma Control Questionnaire (ACQ) (Appendix 1),^32^ (5) being able to understand, read and speak the Dutch language (i.e., based on self-report), and (6) having access to the internet and a smartphone. Exclusion criteria were having a respiratory disease other than asthma or a non-reversible airway obstruction. The only inclusion criteria for HCPs was that they were treating asthma patients who were participating in the study.

### Procedure

#### Patients

Patients with asthma who visited their HCP for an asthma consultation received the patient information letter. One week later, patients were called by their pulmonary nurse to inquire if they were interested in participation and to answer any questions patients might have. When interested, the pulmonary nurse conducted a short screening to determine eligibility. When deemed eligible, a face-to-face appointment between the patient and the pulmonary nurse was scheduled wherein the informed consent form was signed. Also, patients were registered in Astmakompas and were instructed on how to use both Astmakompas and the spirometer. Patients also received a link to the online baseline questionnaire, which they were instructed to complete on the same day. Twelve weeks later, patients completed the post-intervention questionnaire. Table 1 provides an overview of the assessment schedule regarding the questionnaires. Patients who indicated their interest in participating in individual interviews on their informed consent form were contacted by the researchers towards the end of the intervention period (see Interview with patients and HCPs). Patients received gift vouchers worth 25 euros as compensation for participation in the interview. After the study ended, the patients continued their usual asthma treatment at their respective hospitals.

**Table 1.** Questionnaire assessment schedule for patients with asthma.

	Intervention week											
Study measure	1	2	3	4	5	6	7	8	9	10	11	12
Socio-demographic and clinical variables (7 items)	⬤											
ACQ (6 items)	⬤											⬤
RIQ-MON10 (10 items)	⬤											⬤
EQ-5D-5L (6 items)	⬤											⬤
6-item PCAQ (6 items)	⬤											⬤
iPCQ (6 items)	⬤											⬤
iMCQ (5 items)	⬤											⬤
Asthma control monitoring questionnaire (8 items) including spirometer assessment	⬤	⬤	⬤	⬤	⬤	⬤	⬤	⬤	⬤	⬤	⬤	⬤
FIM (4 items)												⬤
CSQ-8 (8 items)												⬤
SUS (10 items)												⬤
Safety (1 item)												⬤

⬤ Gathered using an online self-report questionnaire

⬤ Gathered using the application Astmakompas

*ACQ = Asthma Control Questionnaire, RIQ-MON10= Respiratory Illness Questionnaire-Monitoring 10, EQ-5D-5L = EuroQol 5 dimensions 5 levels, PCAQ = Perceived Control of Asthma Questionnaire, iPCQ = iMTA Productivity Cost, iMCQ = iMTA Medical Consumption Questionnaire, FIM = Feasibility of Intervention Measure, CSQ-8= Client Satisfaction Questionnaire-8, SUS = S*ystem Usability Scale.

#### Health care professionals

At the start of the study, all HCPs involved in the treatment of asthma patients were asked by the principal investigator of the respective hospital to deliver the treatment with Astmakompas to the participating patients. In Amsterdam UMC, no training for HCPs was required as Astmakompas was already part of usual care. In MST, the involved HCPs received instruction manuals of Astmakompas, as well as instructions on how to implement and use Astmakompas in their treatment procedures through a digital meeting with researchers of the LUMC, an experienced pulmonary nurse from Amsterdam UMC, and the company who had developed Astmakompas (SentoMed B.V.).

During the intervention, all involved HCPs were invited to participate in individual in-depth interviews. In case the HPCs wanted to participate, they signed an informed consent form before the interview. The interview was scheduled at least 12 weeks after starting to work with Astmakompas. HCPs received a gift voucher of 100 euros as compensation for participating in an interview, approximating their standard consulting fees. Questionnaires were administered on paper at the end of the interview.

#### Interviews with patients and HCPs

Semi-structured online interviews were conducted by two researchers (LYJ and AZ) at the end of the intervention period. During the first interview, both LYJ and AZ were present, leading to a common agreement on the interview approach. The subsequent interviews were conducted by LYJ. The semi-structured interview guides contained questions on patients’ and caregivers’ experiences and needs regarding Astmakompas, obstacles and facilitating factors for its successful use, and suggestions for improvement that elaborated on the below-described measures (feasibility, acceptability, usability and potential effects). Interviews were held with all involved HPCs of participating patients.

### Intervention

Astmakompas is a Class I CE-certified digital application that consists of a mobile app for patients, a web portal for HCPs, and a CE-certified wireless spirometer connected to the patient app.^33^ The mobile patient app enables patients to weekly monitor their asthma control using a standardized patient-reported outcome measure. The weekly monitoring also includes a spirometer assessment (Spirobank Smart, Medical International Research, Rome, Italy) to assess lung function. The results of these measurements are communicated to the web portal for HCPs, who receive a smart notification when a pre-defined value is exceeded. Finally, the action plan is completed by the patient, in which the patient indicates what symptoms are currently being experienced. These symptoms are linked to their personal digital action plan, which is compiled in advance with the HCP. The action plan aims to provide personalized guidance on asthma management, including medication schedules and emergency procedures, to help individuals manage their asthma symptoms more effectively. Furthermore, patients can easily and conveniently reach their HCP through the chat function, instead of scheduling time-specific phone calls or appointments, with non-critical asthma-related questions and see an overview of previous results.

The web portal for HCPs allows HCPs to monitor their patient's asthma control, as well as having low threshold contact with patients. HCPs are also able to see the monitoring results of patients’ asthma control over time and can use these data as direct input in their consultations. More information and visuals of Astmakompas can be found in Appendix 2.

### Primary outcome measures

#### Feasibility

The perceived feasibility was assessed using the 4-item Feasibility of Intervention Measure (FIM) for both patients and HPCs, using a five-point rating scale ranging from ‘completely disagree’ to ‘completely agree’.^34^ The total score ranges from 4 to 20. A higher score indicates greater feasibility (range 4–20). Patients’ adherence to, and use of, the different components of Astmakompas was assessed using backlog data of the platform. Specifically, how many times patients completed the asthma monitoring questionnaire, spirometer assessment, and action plan. The feasibility of Astmakompas was also discussed in interviews with patients and HCPs.

#### Acceptability

For patients, the perceived acceptability was assessed using the Client Satisfaction Questionnaire-8 (CSQ-8).^35^ Each item was scored on a Likert scale from 1 to 4. The total score ranges from 8 to 32. A higher score indicates greater satisfaction.^36^ For HCPs, the Acceptability of Intervention Measure (AIM) was used to assess acceptability. Items were rated on a 5-point rating scale ranging from ‘completely disagree’ to ‘completely agree’.^34^ The total score ranges from 4 to 20. A higher score indicates greater acceptability. The acceptability of Astmakompas was also discussed in interviews with patients and HCPs.

#### Usability

The system usability scale (SUS) was used to determine the perceived user-friendliness of the intervention for patients, and the web portal for HCPs, respectively.^37^ The SUS is a 10-item questionnaire with a 6-point Likert scale ranging from ‘strongly disagree’ (0) to ‘strongly agree’ (5). The sum of the scores was multiplied by 2.5 to obtain the total score that ranges from 0 to 100. A higher score indicates higher user-friendliness. A usability score higher than 68 indicates above-average usability. The usability of Astmakompas was also discussed in interviews with patients and HCPs.

#### Safety

Patients were asked whether they had encountered any adverse impacts as a result of engaging in Astmakompas (yes/no). If the answer was affirmative, an open-ended question was posed to assess the nature of these effects. The perceived safety of Astmakompas was also discussed in interviews with patients and HCPs.

### Secondary outcome measures

Asthma control was assessed in patients with the 6-item asthma control questionnaire (ACQ).^38^ Each question has seven answer options (0 ‘no impairment’ to 6 ‘maximum impairment’). A total score was created by dividing the sum of the six items by six.^39,40^

Rescue medication use in the past week was assessed with the sixth item of the ACQ. The seven answer options ranged from ‘none’ to ‘more than sixteen puffs/inhalations’.

The Respiratory Illness Questionnaire-Monitoring 10 (RIQ-Mon10) was used to assess asthma-related QoL.^41^ Items are answered on a 4-point Likert scale with higher scores indicative of more complaints or more restraints (range 10–40).^41,42^

General health-related QoL was measured with the EuroQol 5 dimensions 5 levels (EQ-5D-5L).^43^ The score on the five domains can be averaged to describe the patient's health status. In addition, the questionnaire has a visual analogue scale (VAS) to identify how good or bad the current health of the participant is. The VAS ranges from 0 ‘the worst health you can imagine’ to 100 ‘the best health you can imagine’.^44^

Self-efficacy regarding the perceived ability to manage and control asthma was assessed with the 6-item version of the Perceived Control of Asthma Questionnaire (PCAQ). Questions were answered on a 5-point Likert scale with higher scores representing greater perceived ability and confidence to manage and control asthma of more (range 6–30).^45^

Direct costs were assessed with several items of the iMTA Medical Consumption Questionnaire (iMCQ) regarding patients’ asthma-related healthcare consumption in the past three months.^46^ The number of visits to the general practitioner and hospital due to their asthma and how many appointments the patient has had with different practitioners were added up.

Indirect costs were assessed with several items from the iMTA Productivity Cost questionnaire (iPCQ) regarding the impact of disease on absence from work in the past month.^47^ The total amount of absenteeism was calculated by multiplying the number of days of absenteeism and the number of hours per working day of the patient. For respondents who indicated that the absenteeism lasted longer than 4 weeks, the amount of absenteeism was determined in calendar days by calculating the number of days difference between the date on which the iPCQ was completed and the first day of sickness was reported.

All questionnaires used were validated. Permission to use the questionnaires in this study has been obtained from the respective copyright holders. All secondary outcome measures were also discussed in interviews with patients and HCPs.

### Analyses

Descriptive analyses (e.g., M *± SD, N*, percentages) were conducted to summarize the quantitative results. Pre- and post-intervention outcomes were statically compared using paired samples *t*-tests or Wilcoxon signed rank tests. All quantitative analyses were performed in SPSS (SPSS 25.0 statistics for Windows, IBM, Armonk, New York).

The interview audio was transcribed and analysed according to the principles of the Framework Method, guided by the Theory of Acceptance and Use of Technology (UTAUT) model.^48,49^ This iterative analysis process combines inductive and deductive techniques, which matches with the aim of the present research to investigate specific issues regarding patient experiences, as well as discovering additional or unexpected, not a-priori formulated experiences.^48,49^ After familiarization with the transcripts, the first two interviews with patients and the first interview with an HCP were independently analysed and coded by two researchers (LYJ and AZ). After reaching consensus on the coded transcripts, labels and code trees, the remaining interviews from both patients and HCPs were coded by LYJ. Subsequently, horizontal data analysis was performed by LYJ and AZ to explore similarities, differences, and patterns across the perspectives of the patients and HCPs.^50^ Data saturation was achieved concerning patient interviews, whereas all HCPs involved with the patients in this study were interviewed. The interview data were coded using the software Atlas.ti (version 22).

## Results

### Study population

A total of twenty patients were included; however, only 14 patients used Astmakompas and completed the baseline questionnaire. The mean age was about 48 years, and the majority of patients were female and highly educated (see Table 2). The four included HCPs, of whom two pulmonologists and two pulmonary nurses, were all female with a mean age of 44 years (range: 32–59). The working experience as a pulmonologist or pulmonary nurse ranged from 10 to 25 years.

**Table 2.** Demographic characteristics of the patient study population (*n *= 14).

	*n*	%	
Gender			
Male	4	28.6	
Female	10	71.4	
Gender neutral	0	0.0	
Country of birth			
Netherlands	13	92.9	
Other	1	7.1	
Educational level			
Primary education	0	0.0	
Lower vocational education/ preparatory vocational education	0	0.0	
Intermediate general secondary education	0	0.0	
Senior secondary vocational education	5	35.7	
Senior general secondary education/ pre-university education	1	7.1	
Higher professional education	6	42.9	
University education	2	14.3	

	M	SD	Range
Age (years)	47.6	13.0	21–71
Time since asthma diagnosis (years)	24.4	18.4	1–60

M = mean, SD = standard deviation

### Feasibility, acceptability, usability and safety of Astmakompas – quantitative results

**Figure 1.**
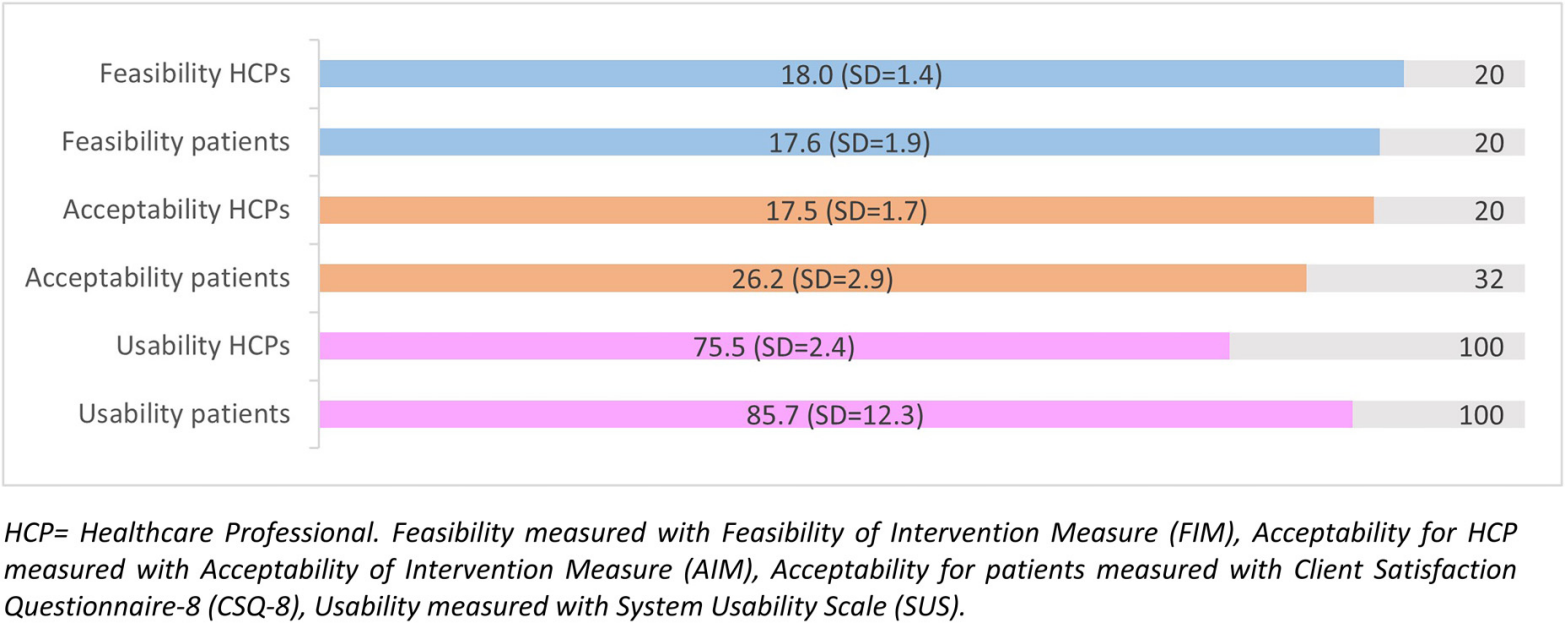
Perceived feasibility, acceptability and usability of the Astmakompas application according to patients and HCPs. Note: *HCP = Healthcare Professional. Feasibility is measured with Feasibility of Intervention Measure (FIM), Acceptability for HCP measured with Acceptability of Intervention Measure (AIM), Acceptability for patients measured with Client Satisfaction Questionnaire-8 (CSQ-8), Usability measured with System Usability Scale (SUS).*

Figure 1 shows the results of the questionnaires regarding feasibility, acceptability and usability. The perceived feasibility and acceptability were scored high by both patients and HCPs, and the perceived usability was scored above average by both. All participants considered Astmakompas to be safe, in terms of not having experienced any adverse effects.

**Figure 2.**
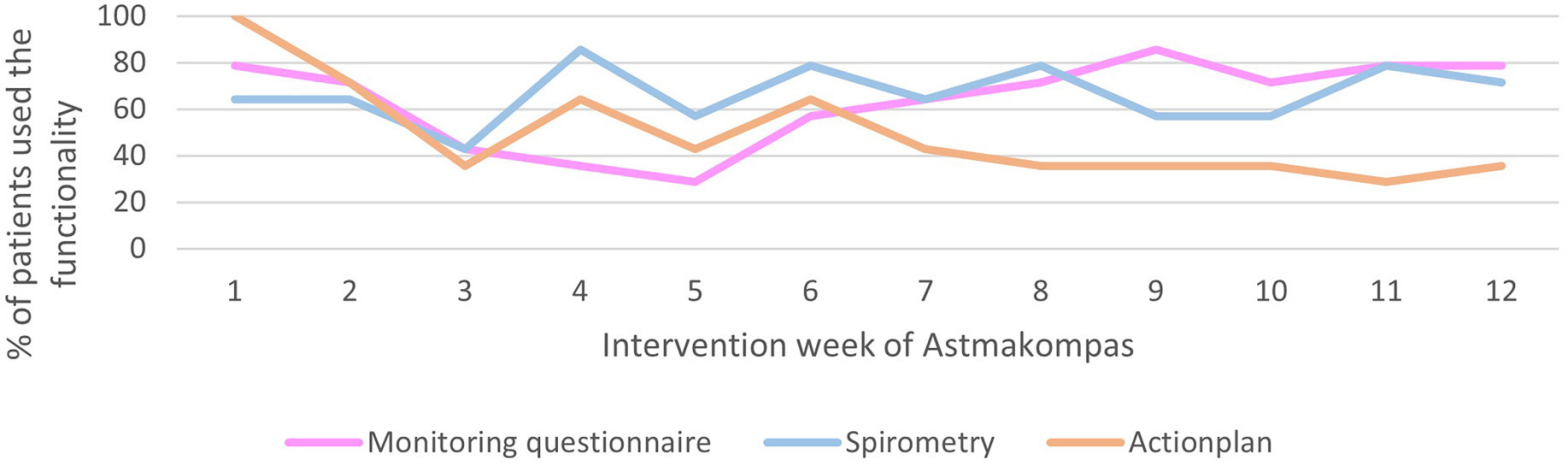
Descriptive information of the use of Astmakompas per week.

Figure 2 shows for each week how many patients used the functionalities of Astmakompas at least once that week. Patients completed the monitoring questionnaire at least once for 7.6 (*SD *= 2.4) weeks and the spirometry measurement for 8.0 (*SD *= 2.9) weeks during the 12-week intervention. One patient completed this every week during the 12 weeks. Both the spirometry functionality and monitoring questionnaire were used irregularly during the intervention. On average, patients used the action plan at least once for 5.9 (*SD = *3.6) weeks, and only one patient completed this every week during the intervention. The use of the action plan seemed to decreased over time.

### Feasibility, acceptability, usability and safety of Astmakompas – qualitative results

A subsample of 11 patients were interviewed, as well as the four HCPs. Five themes emerged from the interviews regarding the perceived feasibility, acceptability and usability.

#### Feasibility of Astmakompas

##### A good explanation and instruction from the pulmonary nurse are essential

Some patients had not used the monitoring overview because they were unaware of its availability. Also, some patients stated they had not used the chat function because they did not know who they would be communicating with, or they assumed that their HCP would be too busy. In addition, some patients were unsure about how often to do the spirometry assessment, and how to do the measurement correctly. Inquiries revealed that in about half of the patients, the HCP had not discussed Astmakompas functionalities and proper use during the consultation. Relatedly, almost all patients expressed a need for more information on how to use Astmakompas at the start of the intervention. Additionally, a few patients expressed a preference for an interim evaluation: ‘*’It would be helpful to discuss your questions after using it for a while, perhaps during an evaluation moment (Patient 9).’’*

##### Astmakompas may help to reduce the workload in the long-term

According to HCPs, Astmakompas is considered compatible with regular care, which typically involves four on-site consultations in addition to an annual asthma check-up in both hospitals. While HCPs emphasized that Astmakompas cannot substitute for the annual asthma check-up, they suggested the potential of Astmakompas to replace on-site consultations, as lung function measurements can be conducted at home. This may help to alleviate the healthcare burden. All HCPs acknowledged that the initial introduction of Astmakompas leads to a rise in patient contact, as patients tend to reach out to their HCP more often during the start of the intervention with intervention- or asthma-related questions. Nevertheless, the ultimate objective of the intervention is to enhance patient self-management in the long term, which may help to reduce the workload for HCPs, and Astmakompas may help to do so. All HCPs indicate that Astmakompas has the potential to reduce their workload in the future when patients’ self-management is improved. One HCP elaborated: ‘*’At a certain point, the patient will recognize the symptoms himself. And as part of patient empowerment, I think that should ultimately lead to that they don't say ‘oh I have more complaints again, doctor, what to do?’ No, they have their own tools to do something with it (HCP1).’’*

#### Acceptability of Astmakompas

##### Astmakompas can lead to better self-management

The general perception regarding Astmakompas’ acceptability was positive, with patients reporting increased awareness of their symptoms and gaining valuable insights into how to manage them effectively. This was particularly notable in terms of increased confidence in recognizing their symptoms, mainly attributed to the spirometry measurement. One patient explained: ‘*'You are really more in touch with your own body, and you really start thinking: Do I feel this right? Am I not feeling this right? Does my experience match the device's readings? It gives some assurance (Patient 6).*'’ In addition, patients indicated that the monitoring overview provided insight into their asthma symptoms. Half of the patients indicated that Astmakompas helped them better control their asthma: ‘*'I increase my medication a little faster now, so I get back into balance faster (Patient 4).* In addition, half of the patients indicated that Astmakompas positively impacted their QoL.

HCPs affirmed that patients seemed to be more aware of their asthma and had an improved understanding of symptoms: ‘*’Patients gain insight and use the action plan to act on their asthma symptoms. In this way, they will also gain confidence in recognizing triggers and adjusting medication accordingly, thereby increasing self-management skills (HCP 1).’’* HPCs perceived an improvement in patients’ abilities to self-manage their asthma and a decrease in patients’ dependency on them: ‘'*See, everything you can manage yourself naturally gives a certain peace and confidence, and that is what I hear. So, that you are less dependent on managing your disease (HCPs 3).’’*

##### Astmakompas should be used customized to care needs

All patients expressed a desire to use Astmakompas for a longer period. Some patients mentioned that they would especially appreciate using Astmakompas in case of monitoring symptoms with new asthma medication and during periods of lower-than-desired lung capacity. Furthermore, half of the patients highlighted that Astmakompas offered a better treatment experience than regular treatment due to symptom insights and the ability to discuss measured data with HCPs during consultations and via chat. However, it was indicated that long-term use should be more tailored to individual requirements: ‘‘*I would like to continue using it because I am more aware of my illness. So, that I can reflect; is it going better or worse than a month ago? So maybe use it on your own initiative (Patient 9).’’* In addition, some patients acknowledged forgetting their weekly measurements or expressed their ability to manage asthma without Astmakompas. HCPs agreed that if asthma control becomes more stable, patients should have the flexibility to use Astmakompas according to the severity of their symptoms.

#### Usability of Astmakompas

##### Astmakompas is user-friendly

All patients praised the user-friendliness of Astmakompas, partly because of the straightforward interface and good app quality. Although a small number of patients encountered initial challenges (e.g., installing the app and connecting the spirometer to the app), they successfully resolved these issues. The different functionalities were generally experienced as easy to use. However, a few patients had difficulty understanding data in the monitoring overview. They suggested several ways to address these challenges, such as a more elaborate explanation of HCPs when starting to use Astmakompas. Furthermore, patients suggested to include instructions on how often to perform the spirometry and the duration of blowing out, as well as a timer feature in the app. All HCPs found the HCP portal user-friendly*.* HCPs described it as clear due to the minimalistic interface.

### Potential effects and usage of Astmakompas

#### Potential effects

The results regarding the pre-post questionnaires can be found in Table 3. Asthma control was improved at post-intervention compared to baseline with a large effect size. Moreover, there was a trend for improvement in QoL with a medium effect size from baseline to post-intervention when measured with the EQ-5D-5L VAS-score. The change from baseline to post-intervention was in the expected direction for rescue medication use, RIQMON-10 score, EQ-5D-5L score, iPCQ and iMCQ with a small to medium effect sizes, but was considered non-significant. No significant changes were found for self-efficacy regarding perceived ability to manage and control asthma

**Table 3.** Data of secondary outcome measures at baseline and post-intervention, along with the results of paired *t*-tests.

	Baseline					Post-intervention					Paired t-test		
	μ	SD	M	IQR	Range	μ	SD	M	IQR	Range	*t*	*p*	Cohen's *d*
Asthma control													
ACQ-6-score	2.1	1.3	2.1	1.5	0–5	1.3	1.0	1.3	1.7	0–3.2	2.61	.02	0.70
Rescue medication use													
Last week additional medication^a^	2.0	1.5	2.0	2.0	0–5	1.4	1.1	2.0	2.0	0–3	−1.63	.10	−0.06
(Asthma-related) quality of life (QoL)													
RIQMON-10-score	22.6	6.9	24.0	7.0	10–33	18.9	7.7	17.0	15.0	10–30	1.70	.11	0.46
EQ-5D-5L-score	1.8	0.7	1.6	1.2	1.0–3.2	1.6	0.6	1.4	1.1	1.0–2.6	1.75	.10	0.47
VAS-QoL	53.5	17.5	56.5	21.0	10–71	67.4	18.9	74.0	20.0	26–95	−2.08	.06	0.56
Self-efficacy regarding perceived ability to manage and control asthma													
PCAQ-score	21.4	2.7	20.5	3.5	17–26	21.6	5.0	22.0	7.3	13–30	−0.231	.84	0.06
(In)direct costs													
iPCQ^b^	34.8	54.7	10	66.5	0–152	22.8	53.3	0.0	22.5	0–152	1.057	.33	0.37
iMCQ-score	4.6	3.7	4.0	7.3	0–10	2.9	3.0	2.5	4.5	0–9	1.52	.15	0.41

^a^ Wilcoxon Signed-ranks test (*Z, p, r*)

^b^ N = 8 (people in paid employment)

Asthma Control Questionnaire-6 (ACQ-6), Quality of Life (QoL), Respiratory Illness Questionnaire-Monitoring 10 (RIQMON-10), EuroQol 5 dimensions 5 levels (EQ-5D-5L), visual analogue scale (VAS), Perceived Control of Asthma Questionnaire (PCAQ), iMTA Productivity Cost Questionnaire (iPCQ), iMTA Medical Consumption Questionnaire (iMCQ).

## Discussion

### Key findings and interpretation

The results of this 12-week pilot study showed that Astmakompas, a supported, digital self-management intervention, was generally perceived as feasible, acceptable, usable and safe by both patients and HCPs in secondary care. The interviews showed that clear instructions for usage and guidance from a pulmonary nurse are paramount. HCPs commented that using Astmakompas in regular care routines could potentially reduce their workload, if patients’ self-management is first improved by the use of Astmakompas. Interview data furthermore suggested customising Astmakompas use to specific care needs of patients, and that Astmakompas could help to improve patient self-management. Finally, this pilot study found improvements over time in asthma control and a trend towards enhanced QoL. The latter results should be interpreted with caution given the small sample, and the lack of a control arm.

The current results align with previous studies highlighting the benefits of digital asthma self-management interventions.^22,27,51,52^ Patients unanimously endorsed the incorporation of Astmakompas to support self-management into usual care, which is in line with the literature emphasizing the importance of increasing patient self-management and providing a tool for HCPs to empower patients.^22,53^ Astmakompas was associated with patients’ improved recognition and management of their asthma symptoms, and promising results were found for QoL. Nevertheless, without a control group, it is difficult to determine whether the observed effect can be attributed to the use of Astmakompas. Previous systematic reviews show similar effects of digital self-management interventions for asthma patients; however, these reviews did find improvements in lung function, rescue medication use and self-efficacy.^27–29^ The latter effects were not found in the current study. Consistent with previous literature,^15,54,55^ some patients, despite meeting the inclusion criteria of uncontrolled asthma, indicated to have good asthma control before the start of the intervention. The perceived asthma control of participants may have contributed to an underestimation of the potential effects and the relatively low intervention adherence.^56^ Moreover, effects on both direct (i.e., healthcare expenses) and indirect costs (e.g., productivity losses) may only become apparent after longer periods of Astmakompas use. Assessing the economic impact of an intervention often requires observing trends and changes over an extended period of time, as it may take time for individuals to experience the full benefits of intervention effects. Long-term studies and continuous monitoring are essential for a comprehensive understanding of the economic outcomes associated with asthma interventions. Nevertheless, these preliminary results are promising for the future implementation of Astmakompas.

Several improvements for Astmakompas were identified, including the patient's need for more comprehensive explanations about how to use Astmakompas and its functionalities. One potential explanation for the patient's need for more information is the potential overestimation of patients’ digital and health literacy by HCPs. Such an overestimation might have caused patients to struggle with properly understanding or applying the given information.^57^ It therefore seems advantageous to give more attention to the preparation and initial phase of using the digital intervention.^58^ For instance, HPCs can go through the app with the patient during the consultation to ensure that all application functionalities are known and understood and intervention goals can be discussed.^59^ Possibly, short interim evaluations could also have a positive effect on the adherence of patients using a digital intervention, since unclarities will be discovered earlier.^59^

Another suggestion for Astmakompas improvement from patients was more flexibility in using the intervention based on one's symptoms and level of control over these. A similar suggestion is reported in a recent review article, highlighting a need for more tailored aspects in asthma self-management interventions. More specifically, to tailor interventions to the individual's needs, health beliefs and abilities, as each individual may need a different approach to address intentional and unintentional barriers that affect treatment adherence.^60^ HCPs mentioned it was important that Astmakompas would be embedded in the existing digital patient systems, which underscores the importance of ensuring a smooth integration of eHealth solutions into existing healthcare processes.^61^

### Strengths and limitations

To our knowledge, this pilot study was the first to investigate a digital, supported self-management intervention for patients with asthma in secondary care. A strength of this study was the recruitment of participants at two hospital sites. Astmakompas was already integrated into usual care at one site, whereas this was not the case for the other site. These different settings allowed for valuable insights into the experiences of HCPs who have been using Astmakompas for some time and new users, which increases the generalizability of the results. Another strength is that the mixed-methods design allowed for a comprehensive understanding of the perspectives and experiences of both HCPs and patients. This study also had some limitations. Although the sample size was considered adequate for the primary and explorative study aims, there is insufficient power to effectively investigate the secondary aims, and the small sample size limits the generalizability.^62^ Additionally, no control group was utilized in this study. Furthermore, purposive sampling was conducted for feasibility reasons, with HCPs playing a significant role in the patient selection. HCPs may have selected patients who seemed more receptive to new technology or self-management support.^63,64^ In addition, patients interested in eHealth might have been more likely to participate, which might have led to an overestimation of the acceptability and usability.

### Implications

There is a need for future longitudinal research to obtain a more comprehensive understanding of the effects in terms of health outcomes and cost-effectiveness. Such research will help to gain insights into the extent to which face-to-face consultations can be replaced by the use of interventions like Astmakompas and in which manner this can be realized. Also, information on feasibility, acceptability and usability after the desired adjustments are made would be beneficial.

### Conclusion

Given the potential of Astmakompas, it demonstrates promise for further implementation and research. Future randomized studies with longer evaluation periods are needed to conclusively assess the impact of this tool on asthma outcomes in patients. However, HCPS must guide the use of the intervention and tailor interventions to meet the individual needs and circumstances of each patient. These findings may also apply to other digital asthma care initiatives in both primary and secondary healthcare settings, with the overarching objective to reduce healthcare burden.

## Supplemental Material

sj-docx-1-dhj-10.1177_20552076241292391 - Supplemental material for Exploring the feasibility, acceptability, usability and safety of a digitally supported self-management intervention for uncontrolled asthma: A pre-post pilot study in secondary care Supplemental material, sj-docx-1-dhj-10.1177_20552076241292391 for Exploring the feasibility, acceptability, usability and safety of a digitally supported self-management intervention for uncontrolled asthma: A pre-post pilot study in secondary care by Anne Zijp, Anke Versluis, Laura Joosse, Taco Kind, Tess MJ Rouveroy van Nieuwaal, Chantal Wassink-Bergman, Ilonka HPAA van Veen, Els JM Weersink, Niels H Chavannes and Jiska J Aardoom in DIGITAL HEALTH

sj-docx-2-dhj-10.1177_20552076241292391 - Supplemental material for Exploring the feasibility, acceptability, usability and safety of a digitally supported self-management intervention for uncontrolled asthma: A pre-post pilot study in secondary care Supplemental material, sj-docx-2-dhj-10.1177_20552076241292391 for Exploring the feasibility, acceptability, usability and safety of a digitally supported self-management intervention for uncontrolled asthma: A pre-post pilot study in secondary care by Anne Zijp, Anke Versluis, Laura Joosse, Taco Kind, Tess MJ Rouveroy van Nieuwaal, Chantal Wassink-Bergman, Ilonka HPAA van Veen, Els JM Weersink, Niels H Chavannes and Jiska J Aardoom in DIGITAL HEALTH

sj-pdf-3-dhj-10.1177_20552076241292391 - Supplemental material for Exploring the feasibility, acceptability, usability and safety of a digitally supported self-management intervention for uncontrolled asthma: A pre-post pilot study in secondary care Supplemental material, sj-pdf-3-dhj-10.1177_20552076241292391 for Exploring the feasibility, acceptability, usability and safety of a digitally supported self-management intervention for uncontrolled asthma: A pre-post pilot study in secondary care by Anne Zijp, Anke Versluis, Laura Joosse, Taco Kind, Tess MJ Rouveroy van Nieuwaal, Chantal Wassink-Bergman, Ilonka HPAA van Veen, Els JM Weersink, Niels H Chavannes and Jiska J Aardoom in DIGITAL HEALTH
