# Supplementary material for: Exploring the feasibility, acceptability, usability and safety of a digitally supported self-management intervention for uncontrolled asthma: A pre-post pilot study in secondary care
Source: Digit Health. 2024 Nov 5;10:20552076241292391. doi: 10.1177/20552076241292391 (PMC11539187; doi:10.1177/20552076241292391)
Supplement: sj-docx-1-dhj-10.1177_20552076241292391 - Supplemental material for Exploring the feasibility, acceptability, usability and safety of a digitally supported self-management intervention for uncontrolled asthma: A pre-post pilot study in secondary care [file sj-docx-1-dhj-10.1177_20552076241292391.docx]

**Appendix 1. Asthma Control Questionnaire (ACQ)**

1. On average, during the past week, how often were you woken by your asthma during the night?
   - Never
   - Hardly ever
   - A few times
   - Several times
   - Many times
   - A great many times
   - Unable to sleep because of asthma
2. On average, during the past week, how bad were your asthma symptoms when you woke up in the morning?
   - No symptoms
   - Very mild symptoms
   - Moderate symptoms
   - Quite severe symptoms
   - Severe symptoms
   - Very severe symptoms
3. In general, during the past week, how limited were you in your activities because of your asthma?
   - Not limited at all
   - Very slightly limited
   - Slightly limited
   - Moderately limited
   - Very limited
   - Extremely limited
   - Totally limited
4. In general, during the past week, how much shortness of breath did you experience because of your asthma?
   - None
   - A very little
   - A little
   - A moderate amount
   - Quite a lot
   - A great deal
   - A vert great deal
5. In general, during the past week, how much of time did you wheeze?
   - Not at all
   - Hardly any of the time
   - A little of the time
   - A moderate amount of the time
   - A lot of the time
   - Most of the time
   - All the time
6. On average, during the past week, how many puffs of short-acting bronchodilator have you used each day?
   - 1-2 puffs most days
   - 3-4 puffs most days
   - 5-8 puffs most days
   - 9-12 puffs most days
   - 13-16 puffs most days
   - More than 16 puffs most days
