# Supplementary material for: Exploring the feasibility, acceptability, usability and safety of a digitally supported self-management intervention for uncontrolled asthma: A pre-post pilot study in secondary care
Source: Digit Health. 2024 Nov 5;10:20552076241292391. doi: 10.1177/20552076241292391 (PMC11539187; doi:10.1177/20552076241292391)
Supplement: sj-docx-2-dhj-10.1177_20552076241292391 - Supplemental material for Exploring the feasibility, acceptability, usability and safety of a digitally supported self-management intervention for uncontrolled asthma: A pre-post pilot study in secondary care [file sj-docx-2-dhj-10.1177_20552076241292391.docx]

**Appendix 1**

**Astmakompas app for patients**

The Astmakompas app enables patients to weekly monitor their asthma control using a standardized patient reported outcome measure. It assesses: shortness of breath, restrictions at work/school/home, extra puffs/inhalations, wheezing and/or cough, night awakening by asthma symptoms, severity of asthma symptoms during morning awakening, and perceived asthma control. Screenshots of the monitoring questionnaire as shown in the Astmakompas application are provided in Figure 1. In the left screenshots, the question is: "How often did you experience wheezing in the past week?" (answer options: never/sometimes/regularly/often/always) and in the second screenshot: "How many nights did you wake up due to your asthma in the past week?" (answer options: never/1 night/2 or 3 nights/4 to 6 nights/every night).


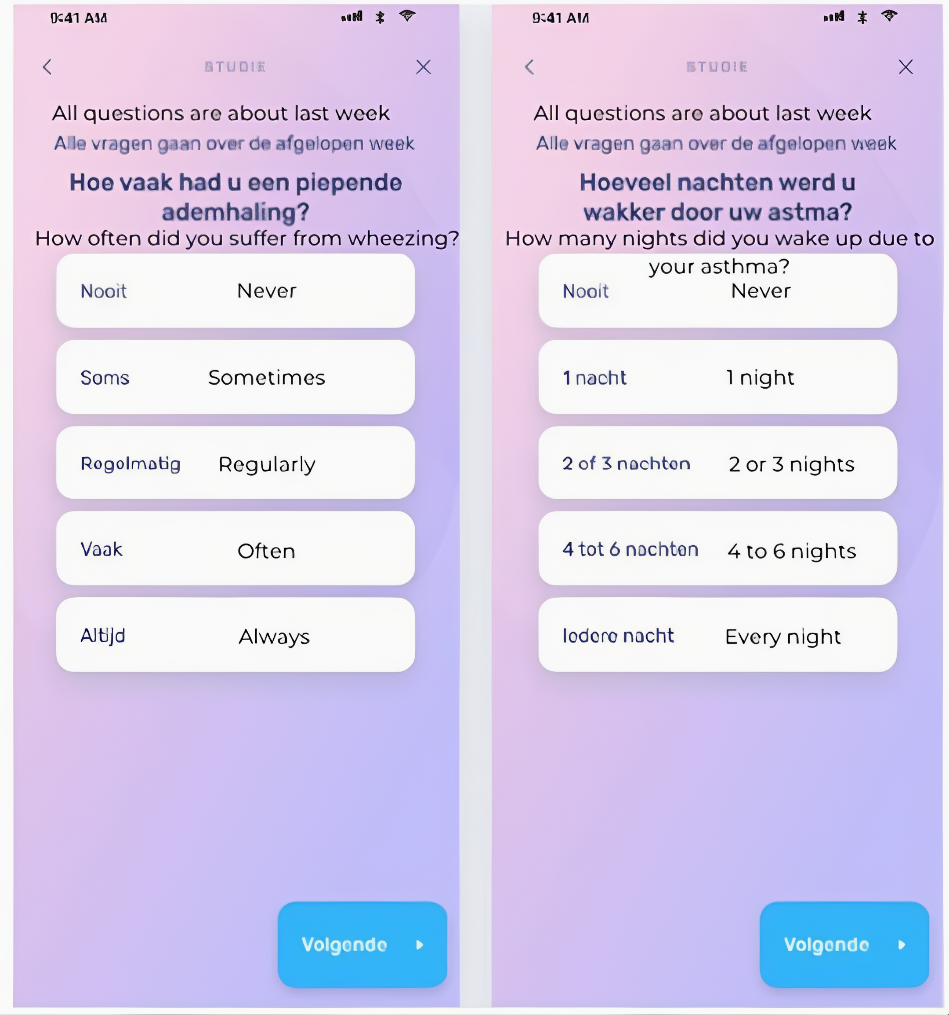


**Figure 1.** Screenshots of a part of the monitoring questionnaire in the Astmakompas application.

Additionally, the weekly monitoring includes a spirometer assessment (Spirobank Smart, Medical International Research, Rome, Italy) to assess lung function. In Figure 2, the steps of spirometry are depicted: the moment when the patient is instructed to begin blowing, from left to right; the moment when the bubble gets larger but the patient must continue blowing; the indication that the patient has blown correctly; and the result of the pulmonary function test. The data were linked to their digital action plan, which was developed at the start of treatment with a pulmonary nurse.
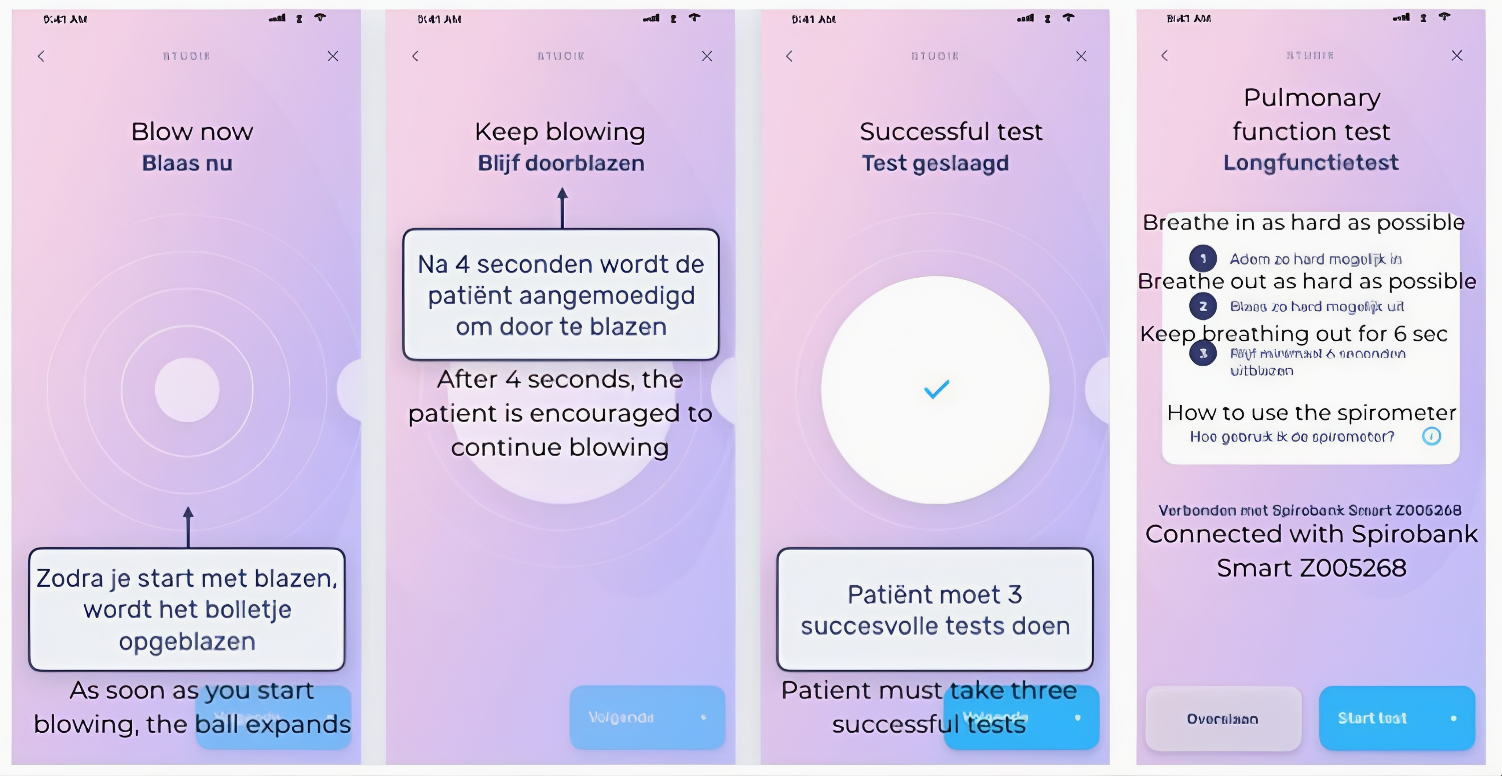


**Figure 2**. Screenshots explaining how to perform spirometry measure.

The action plan aims to increase patients’ awareness of symptoms and the severity of these, as well as its corresponding adverse effects. Specifically, the action plan has four phases that each represent a degree of asthma control, defined by the colours green, yellow, orange, and red (representing good to poor asthma control respectively) (Figure 3). In Figure 3, an example of a yellow action plan is presented, illustrating a deterioration in the patient's condition and the corresponding medications recommended under the yellow action plan. Each phase includes detailed information on when to perform a spirometry measurement, when to increase one’s inhalation medication, how long to continue with the medication, and when and how to seek consultation when increasing the medication dosage is not (sufficiently) effective. The weekly monitoring questionnaire determines the phase the patient is in, thereby educating patients on the appropriate course of action. This early recognition and intervention is aimed to help mitigate adverse health effects and improving health outcomes. Furthermore, patients can easily access their HCP through the chat function with non

critical asthma-related questions.


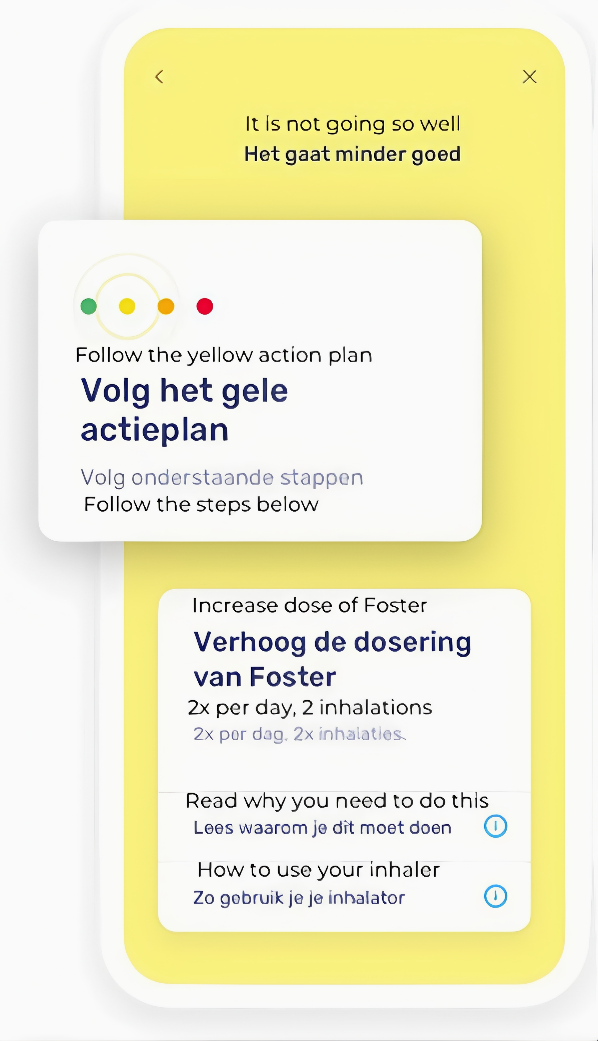


**Figure 3.** Screenshot of action plan in the Astmakompas application.

**Web portal for HCPs**

The web portal for HCPs allows HCPs to monitor their patients’ asthma control as well as having low-threshold contact with patients. When patients’ monitoring results show a deterioration of asthma control (i.e., exceeding a pre-defined threshold value), HCPs receive smart notifications. HCPs are also able to see the monitoring results of patients’ asthma control over time, and can use these data as direct input in their consultations. Furthermore, HCPs can see whether the action plan has been used. Based on the smart notifications and review of the above-mentioned data, HCPs can proactively offer as-needed support. A screenshot of a patient in the HCP portal can be found in Figure 4.


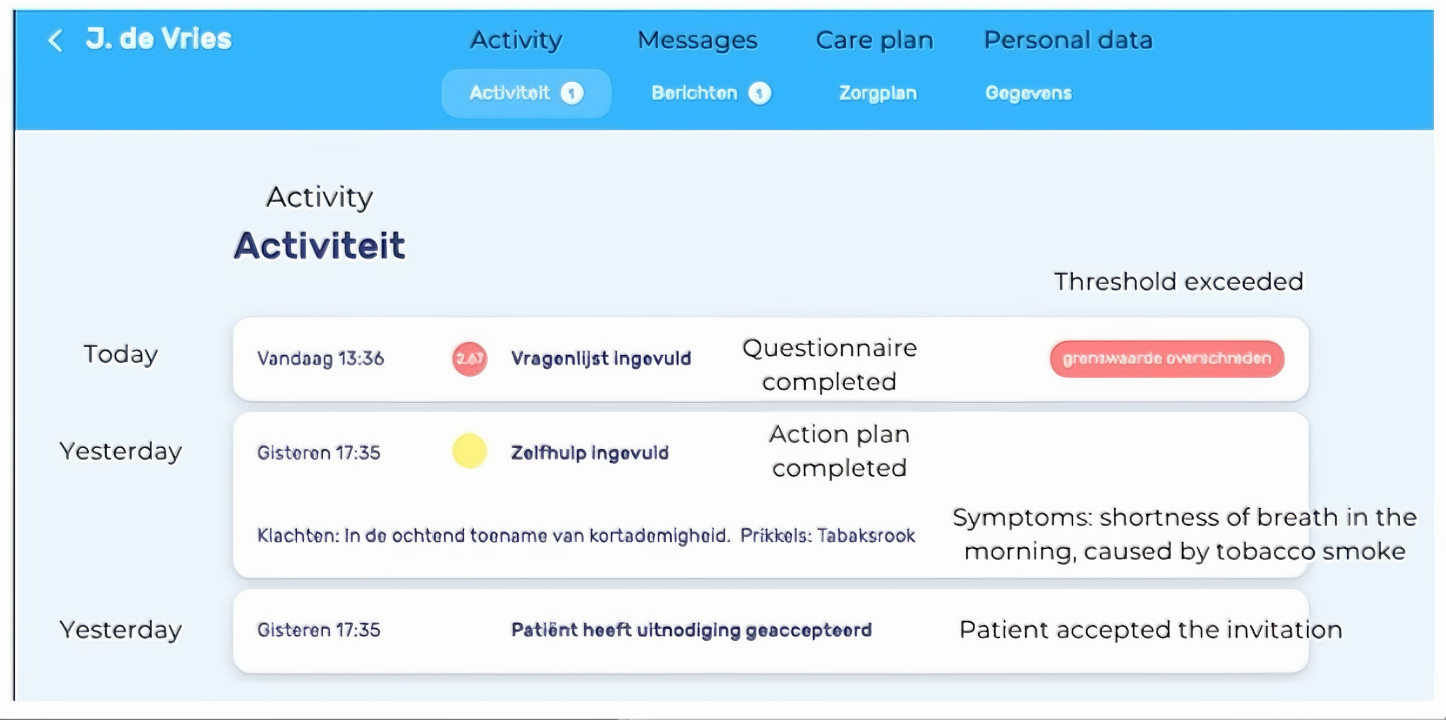


**Figure 4**. Smart notification in case of deterioration of symptoms exceeding a pre-defined threshold value.

The HCPs can read and respond to these messages through the HCP portal (Figure 5). This support is theorized to increase patients’ understanding of asthma and related asthma control. The Astmakompas platform is adaptable to the context of the hospital by means of various clinical and non-clinical configuration options facilitating integration in the local healthcare processes and facilities.


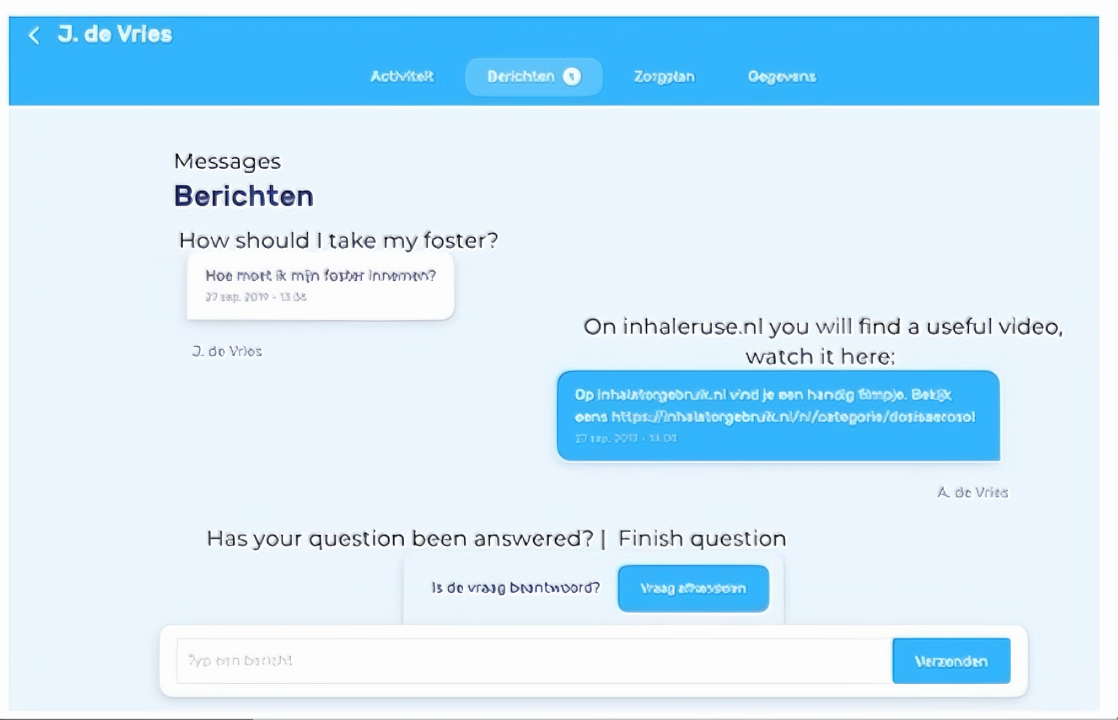


**Figure 5.** Screenshot of communication portal of healthcare professionals.
